# Male and female runners demonstrate different sagittal plane mechanics as a function of static hamstring flexibility

**DOI:** 10.1590/bjpt-rbf.2014.0123

**Published:** 2015-10-06

**Authors:** D. S. Blaise Williams III, Lee M. Welch

**Affiliations:** 1 VCU RUN LAB, Department of Physical Therapy, Virginia Commonwealth University, Richmond, Virginia, USA; 2 Department of Kinesiology and Health Sciences, Virginia Commonwealth University, Richmond, Virginia, USA; 3 B. Young Physical Therapy, Fuquay-Varina, North Carolina, USA

**Keywords:** biomechanics, gender, hamstrings, running

## Abstract

**Background:**

Injuries to runners are common. However, there are many potential contributing factors to injury. While lack of flexibility alone is commonly related to injury, there are clear differences in hamstring flexibility between males and females.

**Objective:**

To compare the effect of static hamstring length on sagittal plane mechanics between male and female runners.

**Method:**

Forty subjects (30.0±6.4 years) participated and were placed in one of 4 groups: flexible males (n=10), inflexible males (n=10), flexible females (n=10), and inflexible females (n=10). All subjects were free of injury at the time of data collection. Three-dimensional kinematics and kinetics were collected while subjects ran over ground across 2 force platforms. Sagittal plane joint angles and moments were calculated at the knee and hip and compared with a 2-way (sex X flexibility) ANOVA (α=0.05).

**Results:**

Males exhibited greater peak knee extension moment than females (M=2.80±0.47, F=2.48±0.52 Nm/kg*m, p=0.05) and inflexible runners exhibited greater peak knee extension moment than flexible runners (In=2.83±0.56, Fl=2.44±0.51 Nm/kg*m, p=0.01). For hip flexion at initial contact, a significant interaction existed (p<0.05). Flexible females (36.7±7.4º) exhibited more hip flexion than inflexible females (27.9±4.6º, p<0.01) and flexible males (30.1±9.5º, p<0.05). No differences existed for knee angle at initial contact, peak knee angle, peak hip angle, or peak hip moment.

**Conclusion:**

Hamstring flexibility results in different mechanical profiles in males and females. Flexibility in the hamstrings may result in decreased moments via active or passive tension. These differences may have implications for performance and injury in flexible female runners.

## Introduction

Running is one of the most popular competitive, recreational, and fitness activities worldwide. In fact, running is a component of, or training modus for, most Olympic and non-Olympic sports. In 2012, roughly 51.4 million Americans ran at least once with approximately 29.4 million of these running at least 50 days per year^1^. The health benefits of running include reducing the risks of (i) chronic disease, (ii) disability, (iii) pain, and (iv) health care costs^2^^-^ 4. However, with the continued popularity of running, there has been a corresponding maintenance in the rate of running-related injuries^5^. The majority of these injuries can be attributed to overuse^3^. As a result, these injuries force an estimated 46% to 65% of runners to stop running and seek medical treatment each year.

The highest risk factor for injuries in runners is weekly mileage. In particular, it is believed that the risk of injury significantly increases as the mileage threshold exceeds 40 miles per week^3^^,^ 6 ^-^ 8. Additionally, higher weekly mileage is correlated with a greater likelihood of muscle tightness, including the hamstrings, which are the most commonly injured multi-joint muscle group in the body^9^^,^ 10. Studies suggest that, as hamstring flexibility decreases, the risk of various running injuries increase^11^, and that there are significant differences in hamstring flexibility between injured and non-injured athletes^12^. Some controversy exists regarding improvement of hamstring flexibility and decreasing risk or incidence of running-related injuries. For example, while studies suggest that increasing hamstring flexibility may decrease the risk or incidence of lower extremity overuse injuries^13^, other studies have demonstrated that hamstring flexibility does not differ between injured and non-injured athletes^14^. Because the methodology between these two studies is not consistent, it is difficult to draw specific conclusions regarding the hamstrings' role in running injury, but it does raise questions regarding the specific effects of hamstring flexibility on running mechanics and injuries. While muscle flexibility may play a role in injury, single anatomical factors are not likely to predict rates or incidences of injuries in runners.

Flexibility has been defined as the ability of muscular tissue to lengthen, given that the articulation travels through the entire movement's span^15^. Lower extremity alignment, with respect to hamstring flexibility and its correlation to risk of injury, has been studied extensively^12^^,^ 14 ^,^ 16. In an open chain, the hamstrings are the primary flexors of the knee, while acting as secondary extensors of the hip. During running, the hamstrings act to slow down hip flexion in the last half of the swing phase (just prior to initial contact) and to extend the hip during the stance phase^17^. Additionally, the hamstrings decelerate tibial extension momentum just before initial contact^18^. Therefore, simultaneous hip flexion and knee extension during late swing result in substantial elongation and eccentric contraction of the biarticular hamstrings, causing extremely high loads during the elongated position of the hamstrings during late swing^19^. Due to energy transfer between phases and the important concentric and eccentric functions of the hamstrings, the flexibility of this group of muscles is not only an important factor influencing running biomechanics, but also a potential factor related to injury during running^18^.

The relationship between hamstring flexibility and injury is poorly understood because the mechanism of tissue damage likely depends on multiple factors, such as joint biomechanics, tissue mechanics, intensity of exercise, fatigue, and tissue structure. It has been shown that simulated hamstring shortening influences gait adversely when the popliteal angle is greater than 15 degrees from full knee extension^20^. These abnormal characteristics were demonstrated by increases in the parameters of walking effort, posterior pelvic tilt, and knee flexion during the stance phase of gait. These were also associated with decreases in walking speed, stride length, step length, hip flexion, pelvic obliquity and rotation, as well as premature ankle dorsiflexion and plantarflexion in stance^20^. While normal hamstring inflexibility would not likely be as extreme, some of these biomechanical effects would result from existing hamstring inflexibility.

In addition to the above kinematic and spatiotemporal characteristics, knee joint moment is another important biomechanical factor that must also be taken into consideration when considering running biomechanics as it relates to static hamstring length. As the hamstring muscles are elongated during late swing prior to initial contact, the moment around the knee is significantly increased. With the hip in 0° extension, maximum knee flexion moment (internal) occurs at full knee extension. With the hip at 90°, there is some variation in position of maximum knee torque with some individuals producing maximum knee torque with the knee near 30-45° and some with the knee at full extension^21^. Furthermore, those with decreased hamstring flexibility exhibit greater knee flexion moment at short muscle lengths and decreased moment at long muscle lengths when compared to individuals with increased hamstring flexibility^22^. Regardless, at initial contact during running, the knee is close to the maximum torque and the hamstring is substantially elongated, resulting is high loads on this muscle during late swing and early stance.

Differences between the sexes may also play a role in running biomechanics. It has been shown that female recreational runners, when compared to males, demonstrate significantly greater peak hip adduction, hip internal rotation, and knee abduction angles. Thus, female runners exhibit significantly different lower extremity mechanics at the hip and knee in the frontal and transverse planes^23^. Additionally, it has been demonstrated that women have less knee flexion angle and more knee valgus angle as well as greater quadriceps activation, and lower hamstring activation as compared to their male counterparts during the stance phase of running, side cutting, and cross cutting^24^. It is unknown whether changes in flexibility of the hamstrings result in different biomechanical profiles in men compared to women.

While hamstring flexibility as it relates to structure and injury is important and has been addressed, there is a lack of research on the differences in running biomechanics in relation to flexible and inflexible individuals. Additionally, while differences in running biomechanics between male and female runners have been investigated, these dissimilarities have not been normalized to account for differences in flexibility due to sex. These differences could help explain how inflexible individuals compensate during running and why injury so often occurs as a result. They could also help explain if differences in male and female running biomechanics are due to sex or inherent flexibility. Therefore, the objective of this study is to compare the effect of static hamstring length on sagittal plane mechanics in male and female runners. We hypothesize that hamstring flexibility will result in similar changes in running mechanics when compared between males and females.

## Method

Individuals in this study were recruited from the University, surrounding communities, and local running clubs, resulting in a sample of convenience of runners who were asymptomatic at the time of data collection. Each subject gave their written informed consent for participation in the study, which was approved by the University and Medical Center Institutional Review Board, Greenville, NC, USA (UMCIRB 10-0437). An a priori power analysis was conducted utilizing data consistent with the variables of interest in the current study (α=0.05, β=0.80). Each variable was used independently for the power analysis, and peak hip angle was found to require the largest number of subjects to obtain significance. Based on this analysis, a sample size of 8 subjects per group was established for comparisons with adequate statistical power. In order to account for attrition and protect from type II error, the study included a total of 40 male and female subjects ranging in age from 18-50 years. Participants were placed in groups based on hamstring length, measured as the number of degrees lacking from zero, where zero is full knee extension with the hip at 90 degrees (popliteal angle). All subjects in this study had hamstrings that were classified as either flexible or inflexible. There were 4 groups consisting of 10 individuals in each group: flexible males, inflexible males, flexible females, and inflexible females sampled from a larger group of 99 runners collected in the current study. All subjects with tight hamstrings had a popliteal angle >29° away from zero. All subjects with flexible hamstrings had a popliteal angle <10° away from zero (Table 1). The values of 10 and 29° were chosen, as they were 1 standard deviation from the mean for the previously mentioned group of 99 runners ranging in age from 29 to 81 years. Participants ran a minimum of 10 miles (16 kilometers) per week for at least 6 months prior to this study. Subjects were excluded from this study if they had any cardiovascular or neurological compromise, current lower extremity musculoskeletal injury, joint replacement, or joint fusion. Runners were not excluded from the study if they had previous lower extremity injuries related to running. 

**Table 1.** Subject demographics.

	**N**	**Age (yrs)**	**Mass (kg)**	**Height (m)**	**Miles/week**	**Popliteal angle (°)**
Flexible Males	10	27.1 (3.7)	76.2 (10.4)	1.80 (0.08)	15.4 (7.5)	4.1 (3.5)
**Inflexible Males**	10	31.7 (8.9)	73.8 (7.0)	1.79 (0.06)	21.0 (11.6)	33.5 (2.6)
**Flexible Females**	10	32.0 (7.6)	64.5 (9.5)	1.67 (0.09)	18.0 (8.0)	3.1 (4.3)
**Inflexible Females**	10	29.2 (5.5)	60.5 (5.3)	1.70 (0.05)	19.1 (12.4)	33.5 (3.9)

Presented in mean (SD).

**Figure 1.**
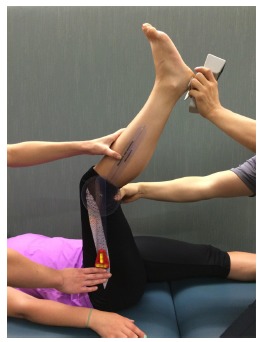
Measurement of hamstring flexibility. Measurements were taken with a goniometer modified with extended arms. The stationary arm was held vertical and in alignment with the upper leg. This was verified with a bubble level. The movement arm was held in line with the fibula extended through the lateral malleolus. A second examiner provided consistent force measured with a handheld dynamometer while examiner one recorded the final angle.

Static hamstring flexibility for both lower extremities was measured by two researchers using a standard goniometer with the subject supine on a mat table. One researcher maintained the knee and hip to be measured in a 90° flexed position and moved the knee into a terminal knee extension position to perform the range of motion measurement. Once terminal knee extension was obtained, the second researcher used a hand-held dynamometer to push the leg being measured with an average force of 10-12 pounds into the patient's end range (Figure 1). The average of 3 measurements was taken for each lower extremity. The contralateral leg remained flat (extended) on mat table during each measurement. All subjects included in the study had symmetrical range of motion (±5°) between right and left limbs. Therefore, only the right limb was utilized in all subjects for comparison between groups. 

**Figure 2.**
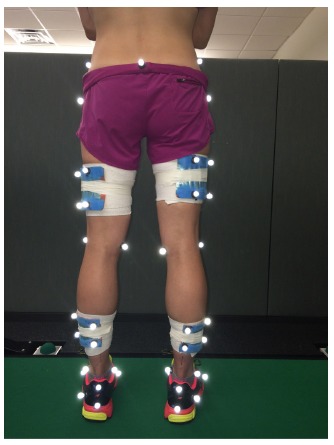
Retroreflective marker placement. A total of 39 markers were placed with at least 3 markers per segment were placed on the pelvis, thighs, shanks, and feet for tracking during running. Static markers were placed over the joints in order to establish anthropometrics and segment coordinate systems.

A three-dimensional running analysis was completed on subjects eligible for participation. A standing calibration trial was collected during which static joint (greater trochanters, medial and lateral knees, medial and lateral maleoli, and medial and lateral forefoot) and segment tracking (calcaneus, shank, thigh, and pelvis) retroreflective markers were placed on bilateral lower extremities (Figure 2)^25^. The static joint markers were used to establish joint centers, segment geometry, and segment coordinate systems. Static markers were removed before the dynamic data collection. During the dynamic data collection, subjects were asked to run along a 16-meter runway at a speed of 3.35 m/s (±5%). Running speed was measured using photocells located 6 meters apart. A fixed running speed was used in order to decrease differences in lower extremity biomechanics and spatiotemporal parameters related to differences in forward velocity. Subjects were instructed to run with their normal running gait. Kinematic data were collected at 240 Hz with a 9-camera motion analysis system (Qualisys^®^ Inc., Glastonbury, CT, USA). Qualisys^®^ software was used to reconstruct 3-dimensional coordinates for each marker. Two force plates (AMTI^®^, Watertown, MA, USA) mounted on the floor of the runway recorded ground reaction forces (GRF) at a sample frequency of 1200 Hz. Kinematic data was time synchronized with GRF data at the time of collection. Subjects were required to run across the force plates for a minimum of 10 successful trials for the right lower extremity. A trial was considered successful if the subject ran with a natural gait over the force plates within the given velocity range while striking at least one of the force plates with their entire right foot. 

Pelvis, thigh, shank, and foot segments were created using Visual 3D Software (C-motion^®^ Inc., Bethesda, MD, USA). Data were analyzed between initial contact and toe-off on the right limb and normalized to 100 data points, with each data point representing 1% of the stance phase of running. A second-order recursive Butterworth filter was used to filter marker data at 12 Hz and GRF data at 50 Hz. For this study, knee motion was defined as the tibia moving relative to the femur, and hip motion was defined as the femur moving relative to the pelvis. Visual 3-D software was used to calculate joint rotations via Cardan sequencing in which motion about the X-axis was defined as flexion/extension at the hip and knee. Joint moments were calculated at the hip and knee. Joint moments were normalized to subject mass and height. Mean joint angle and moment curves were created bilaterally at the hip and knee in the sagittal plane for each group. Peak flexion angles and extension moment values at the hip and knee were calculated. Sagittal plane hip and knee angle at initial contact were also calculated. Data plots were visually assessed for normality and variance homogeneity. Shapiro-Wilk test of normality was used to determine data normality on all variables. Based on the above test, all dependent variables were normally distributed.

Joint angles and joint moments were compared between the groups. These data were analyzed using a 2-factor (sex (df=1), flexibility (df=1), within-subjects (df=36)) analysis of variance (α=0.05) to determine differences between groups for peak knee flexion, peak hip flexion, peak knee extension moment, peak hip extension moment, knee flexion angle at initial contact, and hip flexion angle at initial contact. Post hoc t-tests (α=0.05) were utilized for individual comparisons.

## Results

All results are presented in Table 2. Males demonstrated greater peak knee extension moment than females (M:2.80±0.47, F:2.48±0.61 Nm/kg*m). Inflexible runners demonstrated greater peak knee extension moment than flexible runners (In:2.83±0.56, Fl:2.44±0.51 Nm/kg*m). 

**Table 2.** Dependent variables.

	**Males**		**Females**		**ANOVA (p value)**		
	Flexible (n=10)	Inflexible (n=10)	Flexible (n=10)	Inflexible (n=10)	
Knee					
IC Flexion Angle (º)	12.5 (4.8)	14.8 (3.5)	16.7 (5.9)	14.5 (3.5)	S=0.26 F=0.98 I=0.20
Peak Flexion Angle (º)	43.2 (5.1)	45.1 (6.1)	43.5 (3.2)	45.4 (5.3)	S=0.86 F=0.24 I=0.97
Peak Extension Moment (Nm/kg*m)	2.73 (0.34)	2.86 (0.59)	2.15 (0.49)	2.81 (0.55)	S=**0.05**F=**0.02**I=0.11
Hip					
IC Flexion Angle (º)	30.1 (9.5)‡	31.7 (7.4)	36.7 (7.4)†‡	27.9 (4.6)†	S=0.55 F=0.13 I=**0.03**
Peak Flexion Angle (º)	35.2 (10.3)	37.2 (8.1)	37.1 (7.4)	31.0 (3.2)	S=0.38 F=0.40 I=0.11
Peak Extension Moment (Nm/kg*m)	1.58 (0.41)	1.69 (0.34)	1.76 (0.40)	1.49 (0.23)	S=0.91 F=0.46 I=0.09

S=main effects for sex (df=1); F=main effects for flexibility (df=1); I=interaction (df=36). Values in bold represent significant p values for main effects or interactions from the ANOVA. †post hoc difference between flexible females and inflexible females (p<0.01). ‡post hoc difference between flexible females and flexible males (p=0.05).

**Figure 3.**
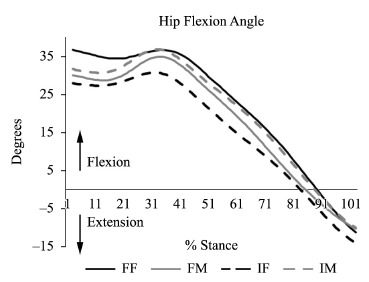
Sagittal plane hip angle during stance. Note that flexible females demonstrate greater hip flexion at initial contact that does not exhibit the same flexion absorption as the other groups. FF=flexible females; FM=flexible males; IF=inflexible females; IM=inflexible males.

A significant interaction existed for hip flexion at initial contact (p=0.03). Specifically, flexible females exhibited more hip flexion than inflexible females (p<0.01) and flexible males (p=0.05) (Figure 3). Interestingly, flexible females not only landed in more flexion but also remained in roughly the same degree of flexion during loading response (Δ=0.4°). 

No differences existed for knee angle at initial contact or peak knee angle. Similar to hip motion, no differences existed for peak hip angle or peak hip moment. 

## Discussion

The purpose of this study was to compare the effect of static hamstring length on sagittal plane mechanics in male and female runners. Mechanical differences existed primarily in flexible females. This is the first study to demonstrate that differences in flexibility result in different mechanical compensations between males and females. This understanding may help define specific interventions for female runners in an attempt to improve performance or reduce injuries. 

At the knee, males exhibited greater peak knee extension moment when compared to females.

While differences in running mechanics have been demonstrated between sexes^23^, the majority of these differences were observed in joint movement (kinematic variables) and in the secondary planes of motion (frontal and transverse). Specifically, females demonstrate significantly greater peak hip adduction, hip internal rotation, and knee abduction angles^23^. Females also demonstrate less knee flexion angle, associated with greater quadriceps activation and lower hamstring activation when compared to males during running and cutting activities^24^. Females are often termed as "quad-dominant" and less able to activate their hamstrings^25^^,^ 26. As running is a series of single-leg landings (squats), the hamstrings are necessary to aid in extension moment at the knee by eccentrically controlling anterior motion of the tibia^4^^,^ 26 ^,^ 27. If females have reduced hamstring activity, this may partially explain the reduction in knee extension moment. This further requires that the knee extension moment be produced by the quadriceps and may place increased stress on the patellofemoral joint, a common injury among female runners^28^. If a runner does not use their hamstrings adequately (magnitude or timing), which may be the case in females, this may explain why females do not produce as much knee extension moment during stance. Further evaluation of hamstring activation in these individuals is necessary to explain this further.

Inflexible runners demonstrated higher peak knee moment than flexible runners. This is consistent with previous work showing that poor hamstring flexibility is associated with higher knee extension moments^4^. As the hamstrings are eccentrically active in controlling flexion of the knee, decreased length of these muscles may result in passive tension and similar control of knee flexion. Therefore, an individual with inflexible hamstrings could demonstrate increased knee extension moment due to the passive tension of this tight group of muscles. Additionally, as hamstring flexibility decreases, the knee extensors may need to counteract the tighter flexor muscles prior to initial contact, further increasing the extension moment at the knee throughout the stance phase.

Flexible females demonstrated the greatest amount of hip flexion at initial contact (Table 2). Interestingly, the females remained in increased hip flexion during loading response but only flexed an additional 0.4º over this time. This, in combination with a large hip extension moment (1.76 Nm/kg*m) results in increased joint stiffness at the hip joint. While not significant, this group demonstrated a similar pattern at the knee where the flexible females flexed approximately 4 degrees less than the other groups. Specifically, flexible females demonstrated the least knee flexion excursion from initial contact to peak (Δ=26.8°). This creates a stiffer knee resulting in less shock attenuation and potential increases in impact forces. We suggest that this passive flexibility results in a need for the female runners to stabilize the hip joint. The question remains as to whether this is a positive compensation based on performance or injuries in this group. While many of the runners in both the flexible and inflexible groups had a history of running injuries, the number of subjects in the current study is not adequate to establish causation. A much larger cohort of runners followed prospectively is necessary to establish strong relationships between hamstring flexibility and lower extremity injuries in runners. 

Previous research has shown that acute changes in hamstring flexibility result in minimal changes in mechanics during running^29^. Limited data exists on mechanical characteristics of runners based on hamstring flexibility, independent of intervention. It would be expected that increased flexibility in runners would result in more hip flexion or knee extension at initial contact. Because females are typically quadriceps dominant, increased quadriceps activity along with decreased hamstring activity should biomechanically result in more hip flexion^26^^,^ 27. It would also seem reasonable to assume that the increased flexibility in these females would result in increased knee extension at initial contact. This may result in changes in stride length or stride frequency. While no such changes were recognized in the current study, further studies may focus on the effect of stretching protocols on stride length and stride frequency or the effect of stride manipulation on lower extremity mechanics (i.e. knee extension moments) as they relate to hamstring flexibility. In the current study, we saw no differences in stride length or frequency, which suggested that the differences in knee moment were related to other factors.

Strengthening and facilitating co-activation of the hamstrings has been shown to increase dynamic control of the knee joint^30^. This would suggest that flexible females may not have good dynamic control of the knee, as there is a lack of activation and/or tension in the hamstrings. Therefore, increasing hip flexion at initial contact could be a neuromuscular compensation, as flexible females attempt to optimize the control of the knee through taking away degrees of freedom at the hip or tightening the muscle by lengthening it over the proximal joint. Further understanding of how males and females respond to stretching or strengthening interventions of the hamstrings is necessary to answer this question.

The current is study is limited by its retrospective nature and the collection of data on a sample of convenience. This study only provides a baseline upon which other randomized, controlled studies can be compared. Further, the subjects in the current study were fairly young and, therefore, not affected by changes in musculoskeletal structure related to aging. It is possible that physiological changes in collagen and neuromuscular control as individuals age may result in further disparity in the biomechanics of running. The risk of type 1 error due to multiple comparisons should be considered in the current study. However, the number of comparisons is relatively small compared to similar biomechanical studies. Further, while there are 6 total comparisons within this study, they are spread across 2 joints (knee and hip), include both kinematics and kinetics, and occur at different times during the stance phase of gait. The lack of control of stride frequency in the current study may also have an impact on the overall utility of the results. However, there were no differences in stride frequency between groups in the current study.

In conclusion, male and female runners respond to landing with different mechanics based on their level of hamstring flexibility. Flexible females demonstrate the lowest knee extension moment and greatest amount of hip flexion, particularly at initial contact. Understanding how these mechanics affect performance and injury patterns may aid in the development of treatment programs focused on strength, increasing passive control, or gait training.
